# Exploring nursing care needs and the role of sleep quality in women undergoing infertility treatment: implications for personalized nursing interventions

**DOI:** 10.3389/fpubh.2025.1674046

**Published:** 2025-10-17

**Authors:** Xiaoyuan Hu, Jinxiao Li, Jingjing Liu, Ningxia Sun, Jing Shi, Xiaohui Zhai, Lingling Li

**Affiliations:** ^1^Reproductive Center, Second Affiliated Hospital of Naval Medical University, Shanghai, China; ^2^Department of Obstetrics and Gynecology, Second Affiliated Hospital of Naval Medical University, Shanghai, China; ^3^Department of Nursing, Fengcheng Hospital, Shanghai, China

**Keywords:** infertility, nursing needs, assisted reproductive technology, sleep quality, multiple linear regression

## Abstract

**Objective:**

To investigate the status and influencing factors of nursing care needs among women undergoing infertility treatment, and to inform personalized and stage-specific nursing strategies.

**Methods:**

A cross-sectional study was conducted among 163 infertile women receiving outpatient treatment at a tertiary reproductive center in Shanghai between December 2024 and February 2025. General demographic and clinical data were collected using structured questionnaires. The Nursing Needs Assessment Scale for Infertility Patients was used to evaluate three dimensions of nursing demands. Differences in needs scores were compared across treatment stages and sleep quality levels. Pearson correlation and multiple linear regression analyses were performed to identify influencing factors.

**Results:**

Across all participants, the total nursing needs score averaged 37.8 ± 7.9 (range: 22–58). Mean dimension scores were 8.20 ± 2.84 for physiological and psychological needs, 15.42 ± 6.47 for information needs, and 4.37 ± 1.88 for support needs. Strong positive correlations were observed among the three dimensions (*r* = 0.65–0.76, all *p* < 0.001). Nursing needs varied significantly across treatment stages: women with ≥2 IVF cycles reported higher total needs (43.54 ± 8.75) than those in ovulation monitoring (35.29 ± 7.12) or first IVF cycle (36.42 ± 7.88; *p* < 0.001). Support needs differed significantly across treatment phases (*p* = 0.037). Sleep quality was also a key determinant: patients with very poor sleep had the highest needs (total score 48.12 ± 6.37) compared with those reporting good sleep (34.16 ± 6.89; *p* < 0.001). Multivariate regression confirmed that poor sleep quality predicted higher physiological and psychological needs (*β* = 1.087, *p* = 0.002), higher educational attainment was associated with stronger information needs (*β* = 1.669, *p* = 0.045), and advanced treatment stage independently predicted higher support needs (*β* = 0.082, *p* = 0.003). The adjusted *R*^2^ of the final model was 0.21.

**Conclusion:**

Infertile women present with moderate to high levels of multidimensional nursing needs, which are significantly influenced by sleep quality, education level, and treatment stage. These findings highlight the importance of dynamic, individualized, and stage-specific nursing interventions to improve patient experience and treatment outcomes in assisted reproduction.

## Introduction

Infertility, defined as the inability to achieve a clinical pregnancy after 12 months or more of regular unprotected sexual intercourse, currently affects approximately 10–15% of couples of reproductive age worldwide (1, 2). With demographic transitions, environmental pollution, and shifting reproductive choices, the global incidence of infertility is steadily increasing, placing substantial physical, emotional, and economic burdens on affected individuals and healthcare systems (3). In China, infertility has become a major public health concern, with an increasing number of couples seeking medical assistance to fulfill their reproductive desires (4, 5).

Assisted reproductive technologies (ART), such as ovulation induction, intrauterine insemination, and *in vitro* fertilization (IVF), have emerged as the cornerstone of clinical management for infertility. Despite technological advances and expanding access, ART remains a complex, prolonged, and psychologically taxing process. Women undergoing infertility treatment are often subjected to repeated hormonal stimulation, invasive procedures, high financial costs, and substantial emotional uncertainty regarding treatment outcomes. These factors collectively contribute to a heightened risk of psychological disorders, including anxiety, depression, and sleep disturbances, which may peak during specific treatment phases (6, 7). Importantly, accumulating evidence suggests that these psychosocial burdens may not only affect patients’ mental health but also reduce treatment compliance, undermine therapeutic efficacy, and adversely impact reproductive outcomes (8).

Within this context, professional nursing support plays an indispensable role. Beyond basic physiological care, infertility patients have expressed growing needs for emotional support, reliable health information, decision-making assistance, and social inclusion (9). Timely identification and fulfillment of these multidimensional nursing needs are essential to alleviating patients’ psychological distress, improving their engagement with treatment, and enhancing satisfaction and outcomes. However, existing studies on infertility care have predominantly focused on medical or psychological aspects, while systematic evaluations of nursing needs remain scarce in the Chinese context. Moreover, few studies have explored how nursing needs evolve across different treatment stages, or how modifiable factors such as sleep quality contribute to these needs (10, 11).

To address these knowledge gaps, this study aims to comprehensively assess the nursing care needs of infertile women and identify key influencing factors. Guided by patient-centered care principles and behavioral health frameworks, we conceptualize nursing needs as dynamic responses shaped by both psychosocial stressors (e.g., sleep disturbance, emotional distress) and treatment-related challenges (e.g., repeated IVF cycles, high financial burden). This theoretical lens allows us to examine not only the prevalence of nursing needs but also the mechanisms through which individual and contextual factors shape them in assisted reproduction. Particular attention is given to treatment stage and sleep quality, which may serve as practical indicators for personalized and dynamic nursing intervention. The findings may contribute to the development of more effective, stage-specific, and patient-centered care models in reproductive medicine.

## Materials and methods

### Study design and participants

This cross-sectional study was conducted at the outpatient infertility clinic of a tertiary reproductive center in Shanghai between December 2024 and February 2025. A total of 163 female patients undergoing infertility evaluation or treatment were recruited using convenience sampling. Recruitment procedures involved consecutive screening of women attending the outpatient infertility clinic during the study period. Of 178 women initially approached, 15 were excluded due to not meeting inclusion criteria (psychiatric illness = 4, communication difficulties = 3, incomplete survey data = 8), resulting in 163 participants included in the final analysis. The participation rate was therefore 91.6%. Inclusion criteria were: (1) diagnosed with primary or secondary infertility according to WHO criteria; (2) currently receiving or preparing for assisted reproductive technology (ART) treatment; (3) aged 20–45 years; and (4) able to provide informed consent and complete the survey independently. Exclusion criteria included major psychiatric illness, communication disorders, or incomplete data.

The required sample size was estimated based on the number of independent variables planned for regression analysis. Assuming 15 predictor variables, a minimum of 8 cases per variable was set, resulting in 120 participants. Allowing for a potential 15% exclusion rate, the recruitment target was set at 163. Post-hoc power analysis indicated that with 163 participants and an effect size of *f*^2^ = 0.15 (medium), the regression model achieved >80% power at *α* = 0.05, supporting adequacy of the sample size.

### General information questionnaire

The general questionnaire was developed by the research team based on a review of the literature and expert group discussions. It was designed to collect sociodemographic and lifestyle data, including age, educational level, occupation, monthly household income, place of residence, religious belief, self-reported sleep quality, exercise frequency, smoking and alcohol use, cause of infertility, and current treatment type. Sleep quality was assessed using a single-item Likert measure (good, fair, poor, very poor), which has been widely used in clinical and epidemiological research for its brevity and feasibility when respondent burden is a concern. To address sparse categories, sensitivity analyses were performed by collapsing levels (good/fair vs. poor/very poor), and the associations remained robust. Additionally, regression models were tested with ordinal coding to account for trends across categories. Although not as comprehensive as validated tools such as the Pittsburgh Sleep Quality Index (PSQI), this single-item measure has demonstrated good convergent validity with multi-item scales in prior studies. To address potential limitations, sensitivity analyses were conducted by collapsing categories (good/fair vs. poor/very poor), and the observed associations with nursing needs remained consistent.

### Chinese version of the infertility nursing needs assessment scale

The original “Nursing Needs Assessment Scale for Infertile Patients” was developed by Park et al. (8) in South Korea. The Chinese version used in this study was translated and culturally adapted by Li et al. (12). In addition to internal consistency, Li et al. (12) reported satisfactory structural validity through confirmatory factor analysis (CFA), supporting the three-factor model of the scale, as well as acceptable test–retest reliability over a two-week interval (ICC = 0.82). Cross-cultural adaptation followed COSMIN guidelines, including forward–back translation and expert panel review, ensuring conceptual equivalence across contexts. The scale comprises 15 items grouped into three dimensions: (i) physiological and psychological needs, (ii) information needs related to infertility treatment, and (iii) support needs. Each item is rated on a 5-point Likert scale ranging from 1 (“not needed”) to 5 (“very much needed”). Total scores range from 15 to 60, with higher scores indicating greater overall nursing needs. In the present study, the Cronbach’s *α* coefficient for the total scale was 0.915, and for each subscale ranged from 0.905 to 0.913, indicating excellent internal consistency.

### Survey administration and data quality control

Participants were recruited from a specialized infertility outpatient clinic. After obtaining informed consent, general information was collected through face-to-face interviews by trained research personnel. The nursing needs questionnaire was administered electronically via a secure WeChat-based survey platform. Participants were provided with instructions and given 20–30 min to complete the form. To ensure data completeness and validity, the electronic survey was programmed with mandatory fields, dropdown menus, and time constraints. After submission, all responses were screened for logical consistency and completeness. Questionnaires with repetitive patterns or inconsistent responses were excluded. A total of 163 electronic questionnaires were distributed, collected, and deemed valid. Although this yielded a 100% effective response rate among included participants, the denominator reflects only those eligible and willing to participate (163/178). Clarification is thus provided to avoid overestimation of response rates.

### Variable definitions and grouping

Participants were grouped according to treatment stage: (1) ovulation monitoring, (2) first-time IVF, and (3) second or more IVF cycles. Sleep quality was self-assessed and categorized as: good, fair, poor, or very poor. These groupings were used to explore variability in nursing needs across clinically relevant subpopulations.

### Statistical analysis

All statistical analyses were conducted using SPSS version 26.0 and Python (for advanced plotting). Continuous variables were tested for normality using the Shapiro–Wilk test and presented as mean ± standard deviation. Categorical variables were summarized as frequencies and percentages. Group differences in nursing needs scores across treatment stages and sleep quality categories were evaluated using one-way ANOVA with *post hoc* pairwise comparisons (LSD or Games–Howell as appropriate). Pearson correlation analysis was performed to examine relationships among the three dimensions of nursing needs. Multiple linear regression analysis was used to identify independent predictors of total nursing needs score, with covariates including age, education level, income, treatment stage, and sleep quality. Although variables with *p* < 0.1 in univariate analysis were initially considered, the final regression model was prespecified based on theoretical and clinical relevance (sleep quality, educational level, and treatment stage) to reduce risks of bias and overfitting. This approach aligns with recommendations for constructing parsimonious, theory-driven models. Regression results are reported with unstandardized coefficients (*β*), standard errors, standardized coefficients, 95% confidence intervals, and adjusted *R*^2^ values. Diagnostic checks (multicollinearity, residual normality, homoscedasticity) confirmed model validity.

## Results

### General characteristics of participants

**Figure 1:**
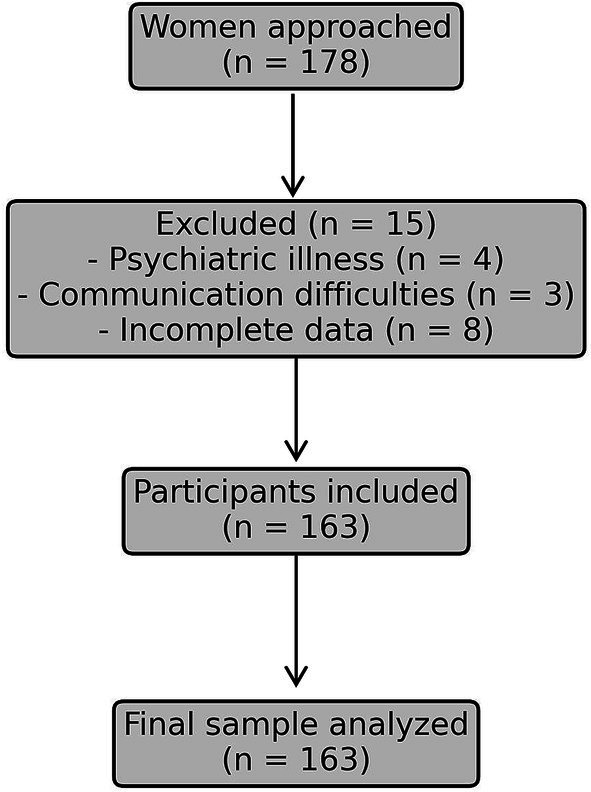
Participant flow diagram.

A total of 163 infertile women were included in the analysis. The participant flow is depicted in Figure 1, illustrating the number approached, excluded, and retained in accordance with STROBE guidelines. The majority were aged 31–40 years (62.5%). Unexplained infertility accounted for 23.9% of cases, while 10.7% had undergone two or more IVF cycles. Detailed demographic and clinical characteristics are presented in Table 1.

**Table 1:** General characteristics and univariate analysis of infertile women (*n* = 163).

Variable	Group	*n*	Physiological and psychological needs	Information needs related to infertility treatment	Support needs
Age	21–30 years	40	8.20 ± 2.83	13.90 ± 5.74	4.42 ± 1.87
	31–40 years	102	8.27 ± 2.90	15.93 ± 6.79	4.38 ± 1.96
	≥41 years	21	7.83 ± 2.68	15.86 ± 5.97	4.19 ± 1.44
*F*			0.231	1.481	0.114
*P*			0.794	0.231	0.892
Educational level	Associate degree or below	50	8.52 ± 3.29	14.24 ± 6.46	4.36 ± 1.95
	Bachelor	90	7.92 ± 2.40	15.59 ± 6.53	4.28 ± 1.84
	Master or above	23	8.57 ± 3.34	17.35 ± 5.93	4.74 ± 1.89
*F*			0.938	1.906	0.552
*P*			0.394	0.015	0.577
Occupation	Labor/business/service	27	8.15 ± 2.64	14.96 ± 6.09	4.70 ± 2.20
	Self-employed/private	29	8.59 ± 3.56	14.90 ± 6.48	4.14 ± 1.89
	Administrative/professional	56	8.25 ± 2.57	17.09 ± 6.41	4.55 ± 1.73
	Unemployed	20	8.70 ± 3.21	15.70 ± 7.36	3.70 ± 1.81
	Others	31	7.45 ± 2.47	13.13 ± 5.80	4.39 ± 1.86
*F*			0.831	2.548	1.100
*P*			0.507	0.040	0.359
Monthly household income	≤10,000 yuan	55	8.36 ± 3.29	14.49 ± 6.27	4.18 ± 1.98
	>10,000 yuan	108	8.12 ± 2.60	15.90 ± 6.54	4.46 ± 1.82
*t*			0.536	−2.317	−0.904
*P*			0.055	0.042	0.889
Place of residence	Urban	112	8.21 ± 2.89	15.46 ± 6.64	4.37 ± 1.86
	Town	36	8.19 ± 2.46	15.92 ± 5.98	4.75 ± 2.06
	Rural	15	8.11 ± 3.47	14.00 ± 6.47	3.40 ± 1.18
*F*			0.018	0.466	2.806
*P*			0.982	0.628	0.043
Religious belief	Yes	6	8.33 ± 2.07	17.50 ± 8.57	4.33 ± 2.25
	No	157	8.19 ± 2.87	15.34 ± 6.40	4.37 ± 1.87
*t*			0.120	0.801	−0.046
*P*			0.346	0.231	0.511
Sleep quality	Good	75	7.52 ± 2.38	15.11 ± 5.59	4.25 ± 1.73
	Fair	73	8.59 ± 2.70	15.51 ± 7.13	4.52 ± 2.00
	Poor	13	8.85 ± 3.93	15.92 ± 7.29	4.15 ± 2.15
	Very poor	2	15.00 ± 5.67	21.00 ± 9.90	4.50 ± 0.70
*F*			6.551	0.581	0.310
*P*			<0.01	0.628	0.818
Exercise frequency	Daily	16	7.81 ± 3.17	15.06 ± 6.57	4.06 ± 2.11
	3–5 times/week	24	7.88 ± 2.47	15.75 ± 7.32	4.71 ± 1.65
	Occasionally	101	8.03 ± 2.61	14.97 ± 6.14	4.39 ± 1.89
	Never	22	9.59 ± 3.67	17.41 ± 6.95	4.14 ± 1.89
*F*			2.129	0.892	0.515
*P*			0.099	0.447	0.672
Smoking	Yes	4	7.25 ± 1.89	10.50 ± 4.04	4.50 ± 2.52
	No	159	8.22 ± 2.86	15.55 ± 6.48	4.36 ± 1.87
*t*			−0.674	−1.548	0.142
*P*			0.406	0.392	0.628
Alcohol consumption	Yes	8	7.38 ± 1.51	15.00 ± 6.12	5.38 ± 2.20
	No	155	8.24 ± 2.89	15.45 ± 6.50	4.32 ± 1.85
*t*			−0.838	−0.189	1.564
*P*			0.148	0.410	0.202
Cause of infertility	Male factor	6	9.67 ± 3.98	22.33 ± 6.38	6.67 ± 1.63
	Female factor	35	7.89 ± 2.60	13.54 ± 6.75	4.40 ± 2.09
	Both	82	8.50 ± 3.16	16.02 ± 6.32	8.50 ± 3.16
	Unknown	40	7.63 ± 1.98	14.80 ± 5.83	3.68 ± 1.40
*F*			1.544	3.820	5.429
*P*			0.205	0.110	0.001
Current treatment	Ovulation monitoring	19	8.37 ± 3.08	16.63 ± 5.94	4.63 ± 1.57
	Artificial insemination	8	8.50 ± 5.17	17.00 ± 7.46	4.13 ± 2.03
	First IVF cycle	37	7.86 ± 2.21	14.51 ± 6.31	4.11 ± 1.85
	Medication phase	22	7.64 ± 3.47	13.18 ± 5.80	3.86 ± 1.46
	Oocyte retrieval done	29	7.79 ± 2.74	15.62 ± 6.71	4.66 ± 2.16
	Embryo transfer done	30	8.40 ± 2.19	16.00 ± 6.27	4.23 ± 1.92
	≥2 IVF cycles	18	9.56 ± 3.28	16.78 ± 7.57	5.11 ± 2.00
*F*			1.067	0.926	1.082
*P*			0.385	0.478	0.037

Data are represented as Mean ± Standard Deviation.

### Total nursing needs score

Across all participants, the total nursing needs score averaged 37.8 ± 7.9, with a median of 36 (IQR: 32–43) and a range of 22–58. By treatment stage, women with ≥2 IVF cycles reported the highest needs (43.54 ± 8.75), compared to those in ovulation monitoring (35.29 ± 7.12) and first IVF cycle (36.42 ± 7.88; all *p* < 0.001). By sleep quality, patients reporting very poor sleep scored 48.12 ± 6.37, versus 34.16 ± 6.89 among those with good sleep (*p* < 0.001).

### Univariate analysis of nursing needs dimensions

The mean scores of the three nursing need dimensions were 8.20 ± 2.84 for physiological and psychological needs, 15.42 ± 6.47 for information needs, and 4.37 ± 1.88 for support needs. Univariate analysis of demographic and clinical characteristics is summarized in Table 1. Age was not significantly associated with any dimension of nursing needs (all *p* > 0.05). Educational level showed a significant effect on information needs (*p* = 0.015), with participants holding a master’s degree or above reporting the highest scores. Occupation was also associated with information needs (*p* = 0.040), as administrative/professional participants scored higher than those in other categories. Monthly household income was positively related to information needs, with women reporting >10,000 yuan per month showing higher scores than those with lower income (*p* = 0.042). Place of residence was significantly associated with support needs, with urban and town residents reporting higher scores than rural residents (*p* = 0.043). Religious belief, smoking, and alcohol consumption were not significantly related to any nursing need dimension (all *p* > 0.05). Sleep quality significantly influenced physiological and psychological needs, with poorer sleep associated with higher scores (*p* < 0.01). Exercise frequency was not significantly associated with nursing needs, although a trend toward higher physiological and psychological needs was observed in women who never exercised (*p* = 0.099). Cause of infertility was significantly associated with support needs (*p* = 0.001), with the highest scores reported among women with male factor infertility. Information needs showed a borderline association with infertility cause (*p* = 0.110), whereas no significant difference was observed for physiological and psychological needs. Current treatment stage was significantly associated with support needs (*p* = 0.037), with the highest scores observed in women undergoing two or more IVF cycles. No significant associations were found between treatment stage and the other two need dimensions.

### Multivariate regression analysis of influencing factors

Multivariate linear regression identified sleep quality as an independent predictor of physiological and psychological needs (*β* = 1.087, 95% CI [0.40–1.77], *p* = 0.002). Educational level significantly influenced information needs (*β* = 1.669, 95% CI [0.02–3.32], *p* = 0.045). Current treatment stage was a key determinant of support needs (*β* = 0.082, 95% CI [0.03–0.39], *p* = 0.003). The adjusted *R*^2^ of the final model was 0.21, indicating modest explanatory power. Diagnostic checks confirmed the absence of multicollinearity, normally distributed residuals, and homoscedasticity, supporting the robustness of the regression models (Table 2).

**Table 2:** Multiple linear regression analysis of influencing factors among infertile women (*n* = 163).

Dimension	Variable	Regression coefficient	Standard error	Standardized coefficient	*t*	*P*	95% CI
Physiological and psychological needs	Constant	4.855	4.153	-	1.169	0.244	[−3.32, 13.03]
	Sleep quality	1.087	0.347	0.261	3.131	0.002	[0.40, 1.77]
Information needs related to infertility treatment	Constant	1.505	9.589	-	0.157	0.876	[−17.5, 20.5]
	Educational level	1.669	0.859	0.168	1.942	0.045	[0.02, 3.32]
Support needs	Constant	5.682	2.814	-	2.019	0.042	[0.13, 11.24]
	Current treatment	0.082	0.084	0.081	2.977	0.003	[0.03, 0.39]

Regression coefficients are reported with 95% confidence intervals. Adjusted *R*^2^ of the final model = 0.21.

### Correlations among nursing need dimensions

**Figure 2:**
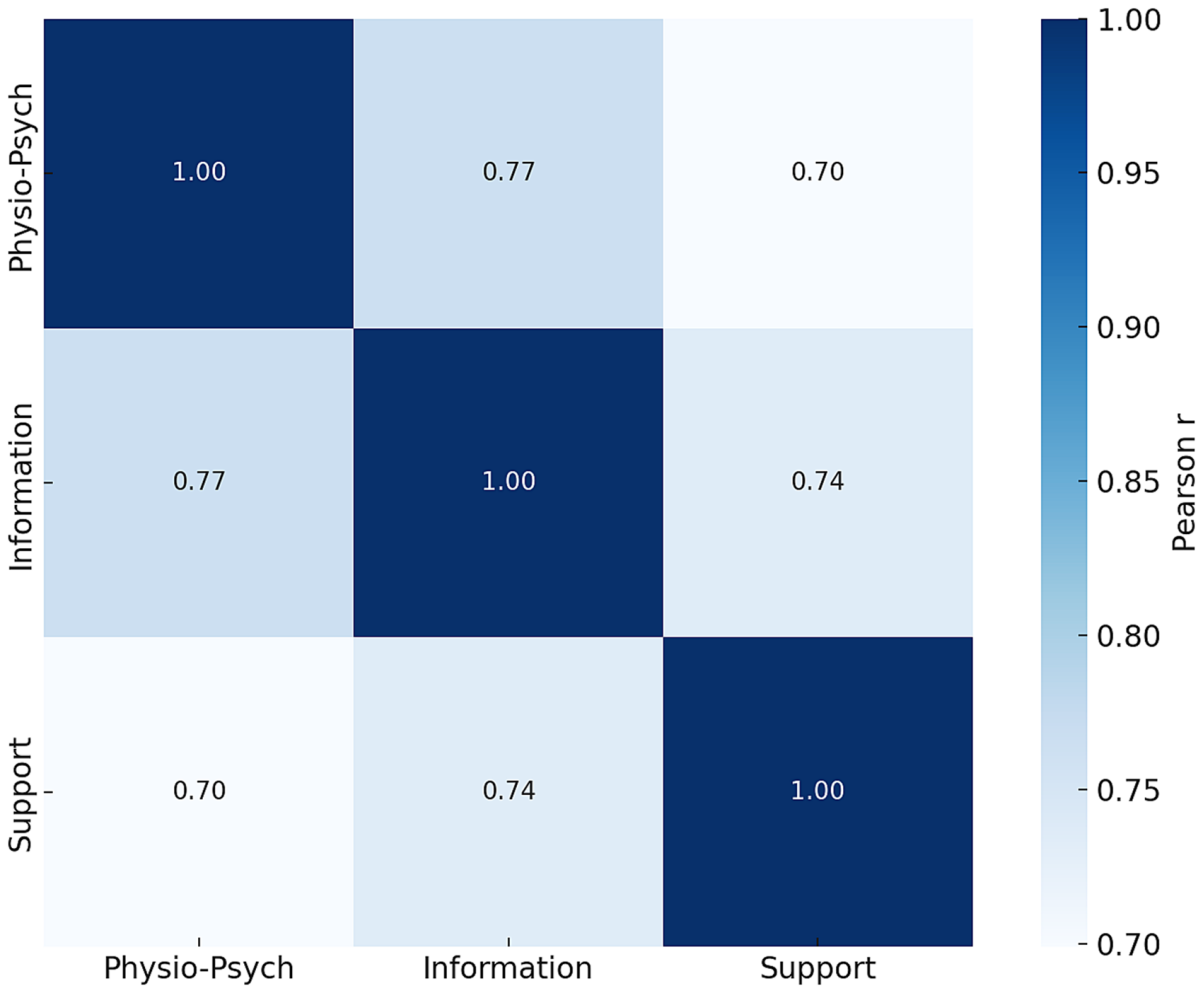
Pearson correlation heatmap among three dimensions of nursing needs. The heatmap displays correlation coefficients (r values) between physiological and psychological needs, information needs, and support needs. Color intensity reflects the strength of the correlations, with darker blue indicating stronger positive associations. All correlations were statistically significant (*p* < 0.001), suggesting substantial co-occurrence among the three domains of need.

Pearson correlation analysis revealed strong positive associations among the three dimensions of nursing needs. Specifically, physiological and psychological needs correlated with information needs (*r* = 0.72), and with support needs (*r* = 0.65). Information needs were also significantly correlated with support needs (*r* = 0.76). All correlations were statistically significant (*p* < 0.001), indicating a high degree of consistency across care domains (Figure 2).

### Differences in nursing needs across treatment stages

**Figure 3:**
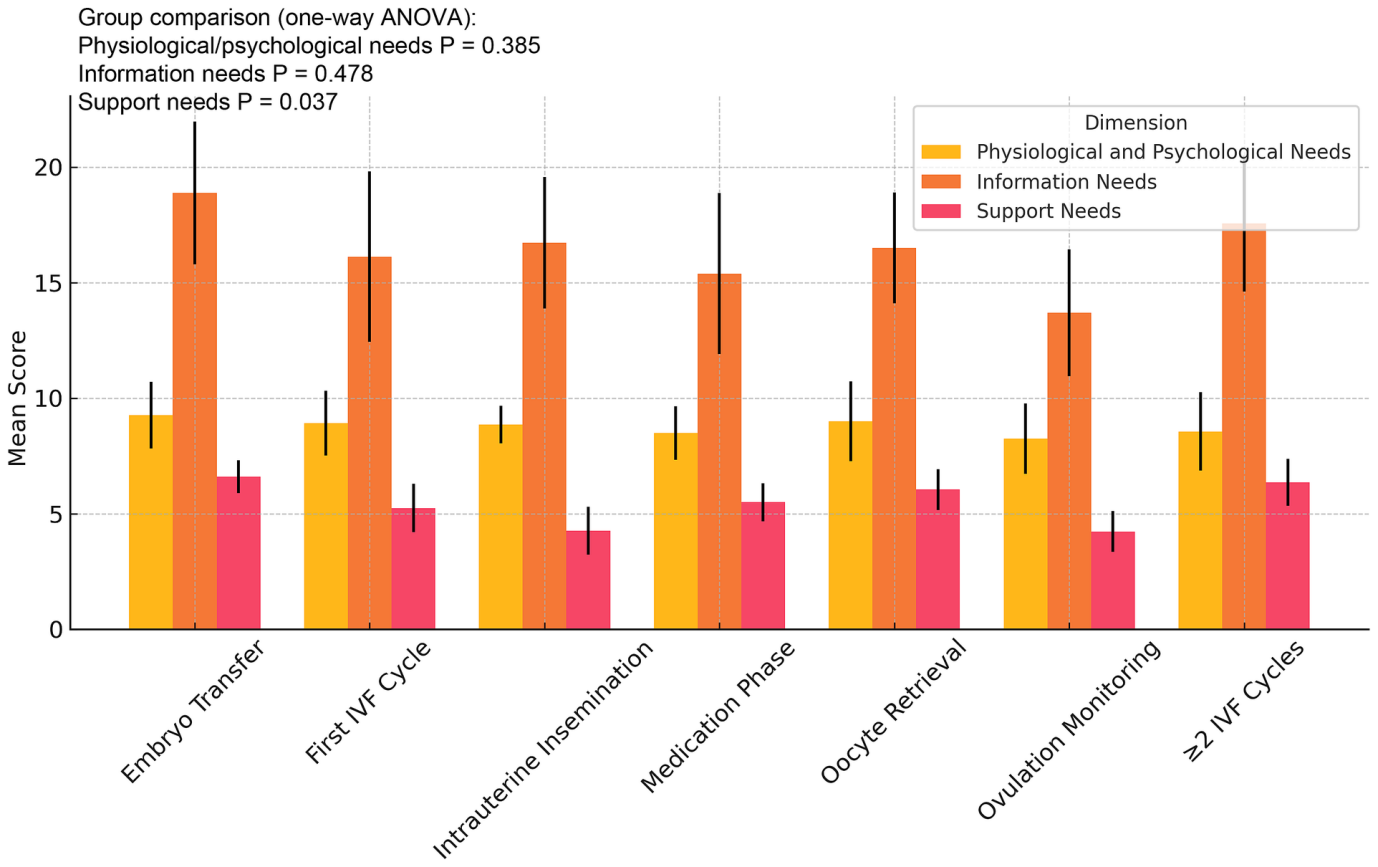
Nursing needs scores across treatment stages in infertile patients. Average scores for physiological and psychological needs, information needs, and support needs are presented across seven treatment stages. Nursing needs increased with treatment progression and peaked among patients undergoing multiple IVF cycles, highlighting the importance of stage-specific nursing support.

As illustrated in Figure 3, the mean scores of the three nursing need dimensions varied across treatment stages. Overall nursing needs tended to increase as treatment progressed, reaching a peak in women who had undergone two or more IVF cycles, reflecting the cumulative physical and psychological burden of repeated procedures. Statistical testing showed that only support needs differed significantly across treatment stages (*p* = 0.037), with the highest scores observed in women with multiple IVF cycles. In contrast, no significant differences were found for physiological and psychological needs (*p* = 0.385) or information needs (*p* = 0.478). These results highlight that while the overall trend suggests higher demands in advanced treatment stages, only the support dimension showed statistically significant variation, underscoring the importance of tailored psychosocial and practical support in later treatment phases.

### Impact of sleep quality on nursing needs

**Figure 4:**
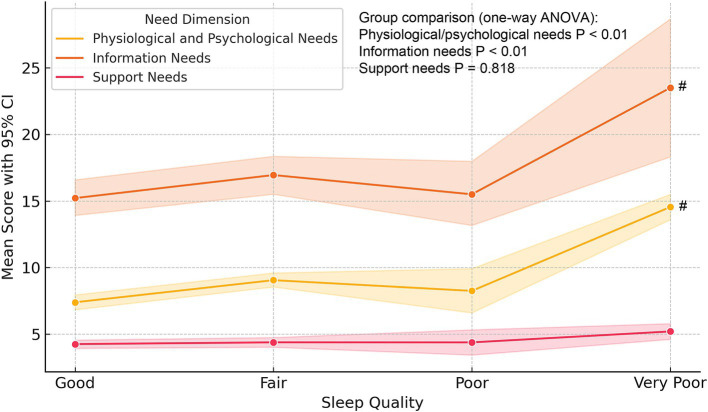
Trends in nursing needs across sleep quality categories in infertile patients. Line plots illustrate the mean scores and 95% confidence intervals for three nursing need dimensions—physiological and psychological needs, information needs, and support needs—across four levels of self-reported sleep quality. A consistent upward trend is observed, with markedly higher scores among patients reporting very poor sleep, indicating that diminished sleep quality is associated with elevated and multidimensional nursing care demands. #: compared with the Good group, *p* < 0.05.

As shown in Figure 4, poorer self-reported sleep quality was consistently associated with higher nursing needs across all three dimensions. Patients with “very poor” sleep had significantly elevated scores for physiological and psychological needs (mean = 14.54) compared to those with “good” sleep (mean = 7.38). Similar trends were observed for information and support needs. These findings suggest that poor sleep exacerbates both physical discomfort and emotional vulnerability, increasing reliance on nursing care. Sleep quality should therefore be integrated into clinical assessments, with targeted interventions aimed at improving both rest and overall well-being.

## Discussion

### Infertile women exhibit high levels of nursing needs

This study highlights the substantial nursing needs among infertile women undergoing assisted reproductive technology (ART), advancing existing literature in two key ways. First, unlike most prior studies that separately addressed psychological burden or informational demand, we employed a multidimensional patient-centered care framework to capture physiological, informational, and support needs in a single model. Second, we uniquely integrated treatment stage and sleep quality as predictors, offering novel insight into how modifiable psychosocial factors interact with treatment progression to shape patient experiences. Given the cross-sectional design, our findings indicate associations rather than causal predictions; for example, poor sleep quality was associated with greater nursing needs, but longitudinal studies are required to confirm temporal direction. The average scores for physiological and psychological needs (8.20 ± 2.84), treatment-related information needs (15.42 ± 6.47), and support needs (4.37 ± 1.88) were all at moderate-to-high levels, consistent with previous studies by Naab et al. (13). These findings suggest that despite growing familiarity with ART, patients continue to experience strong demands for tailored nursing care and professional guidance. Similar findings have been reported in other Asian countries. For example, a Korean study by Park et al. (8) demonstrated that infertile women exhibited comparable multidimensional nursing needs, particularly in information-seeking and emotional support. In Japan, research on infertility-related stigma also highlighted the persistent psychological distress and demand for professional guidance among women undergoing ART (6). These regional similarities suggest that infertile women across East Asia may share common care needs shaped by cultural expectations, family-oriented social norms, and the high emotional investment in childbearing. While Korean and Japanese studies have emphasized stigma, information-seeking, and emotional distress, our findings add originality by quantifying the role of sleep quality—a rarely examined but modifiable factor—in amplifying multidimensional nursing demands. This perspective expands the understanding of patient-centered care in Asian infertility contexts. The rising demand may be attributed to improvements in medical technology, enhanced psychological adaptability, and increased health literacy. While infertility is often associated with anxiety, depression, and negative self-perception (14), recent improvements in public awareness and expanded access to medical information have led to a more rational understanding of infertility and greater confidence in treatment. Although studies have shown that infertile women experience greater psychological stress than those with natural pregnancies (15), others have reported overall good mental health and even lower depression scores than in general female populations (16, 17). These differences may be due in part to widespread dissemination of reproductive health knowledge via media and digital platforms. Given this context, nurses should recognize that infertile women have strong engagement and information-seeking behaviors. Comprehensive, multi-channel health education and psychological support are essential to promoting patient adherence and optimizing clinical outcomes.

### Poor sleep quality is associated with increased physiological and psychological nursing needs

Poor sleep quality is a prevalent yet often under-recognized issue in women undergoing fertility treatment. Previous studies, including that by Chen et al. (18), have shown that women with poor sleep experience more pronounced physiological and psychological distress, leading to elevated nursing demands. Lin et al. (19) also emphasized the critical link between sleep disorders and psychological burden in this population. Patients undergoing IVF frequently report symptoms such as bloating, breast tenderness, dizziness, nausea, and diarrhea, which can further disrupt sleep and amplify anxiety and depression. Meta-analyses have confirmed a significant correlation between infertility and poor sleep quality (20, 21). These findings underscore the need for sleep assessments in nursing practice. Interventions such as psychological counseling, behavioral therapy, and sleep hygiene education may help alleviate treatment-related distress, improve compliance, and ultimately enhance patient well-being.

### Higher educational attainment is linked to greater demand for treatment-related information

Educational level emerged as a significant predictor of information needs in this study. Women with higher education levels demonstrated stronger demands for treatment-related knowledge, likely due to their superior ability to seek, evaluate, and comprehend medical information. Prior studies have indicated that well-educated patients tend to be more proactive in disease management and more psychologically resilient during ART (22, 23). They are also more likely to receive emotional and informational support from their social networks (24). In contrast, women with lower educational attainment may have limited health literacy and face barriers in understanding medical terminology and processes, which could hinder communication and reduce adherence (25, 26). Tailored communication strategies are therefore critical: simple, visual, and jargon-free materials should be used for patients with low literacy, while more detailed and technical explanations may be appropriate for those with advanced education.

### Patients undergoing active treatment report higher support needs

Support needs were highest among women actively undergoing treatment, particularly those in advanced stages such as oocyte retrieval or embryo transfer. These findings reflect the intensified psychological burden and heightened uncertainty experienced during these critical periods. Prior research has shown that patients require clear guidance, emotional reassurance, and individualized support throughout the treatment process (27–29). For example, during ovulation monitoring and timed intercourse, patients often express confusion about procedures and anxiety over outcomes. Intrauterine insemination (IUI) introduces additional technical concerns and information needs. During IVF, especially after oocyte retrieval and embryo transfer, patients frequently encounter physical discomfort and emotional volatility. Studies have reported that psychological support during the post-transfer period may positively influence pregnancy outcomes (30, 31). Furthermore, patients desire structured information on treatment risks, coping strategies, and failure management (32). Nurses should therefore adopt a phase-specific approach, delivering anticipatory guidance and emotional support tailored to the patient’s current stage. Establishing trustful relationships, enhancing bidirectional communication, and involving patients in decision-making can significantly improve satisfaction, compliance, and reproductive outcomes.

### Strengths and limitations of this study

This study has several strengths. First, it is among the few investigations in China to integrate both treatment stage and sleep quality as predictors of nursing needs, offering a more comprehensive understanding of influencing factors. Second, the use of a validated, culturally adapted nursing needs assessment scale ensured reliability and comparability of results. Third, a high response rate (100%) minimized selection bias and enhanced the robustness of findings. However, several limitations should be acknowledged. The cross-sectional design prevents causal inferences, and all participants were recruited from a single tertiary center in Shanghai, which may limit generalizability to other regions. Self-reported sleep quality may also be subject to recall or reporting bias. We acknowledge that a single-item measure cannot fully capture the multidimensional construct of sleep quality. However, it offered a pragmatic approach in a busy outpatient setting and yielded results consistent with the broader literature. Future studies should consider using validated instruments such as the PSQI to provide more comprehensive sleep assessments. Furthermore, although multiple regression identified significant predictors, residual confounders cannot be excluded. Future research should employ longitudinal, multicenter designs with larger and more diverse populations to validate and expand on these findings.

**Figure 5:**
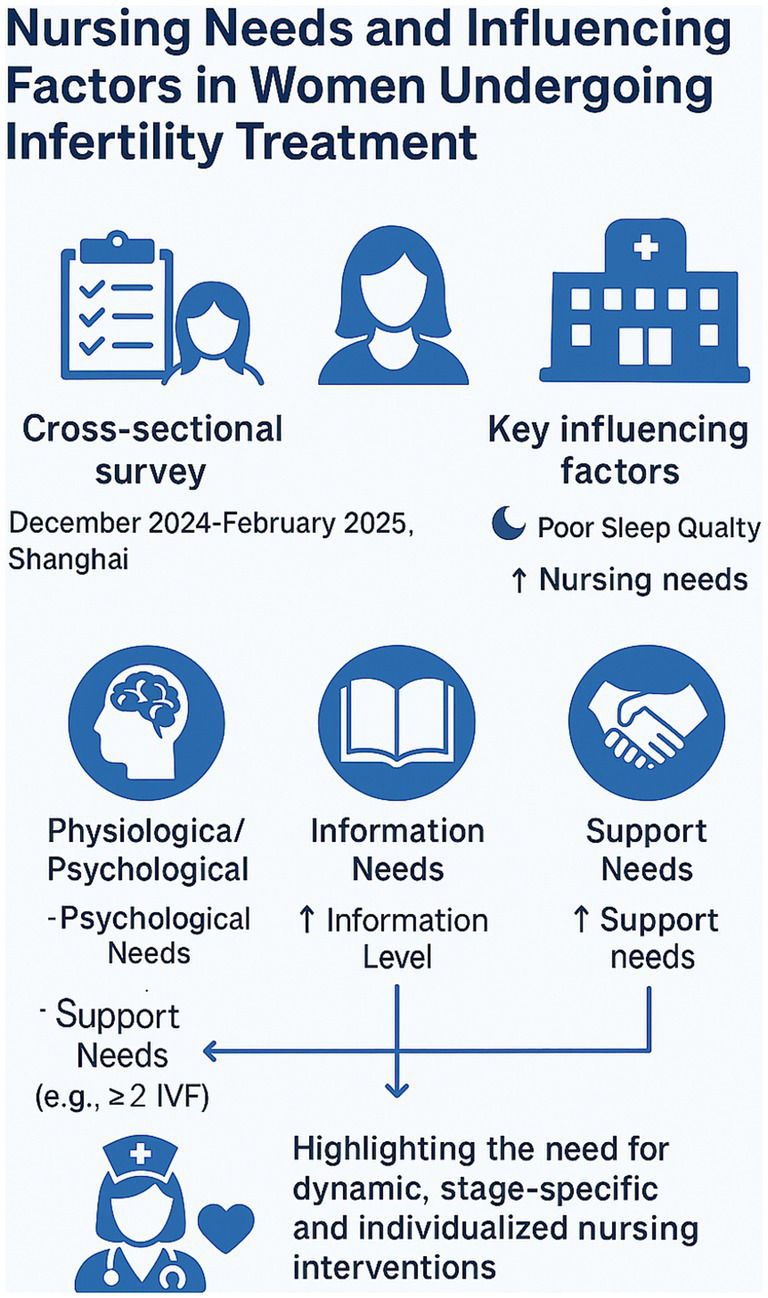
Graphical summary illustrating the nursing care needs of infertile women undergoing ART and the key influencing factors. A cross-sectional survey identified multidimensional nursing demands across psychological, informational, and support domains, with sleep quality, education level, and treatment stage as major predictors. The findings emphasize the necessity of dynamic, personalized nursing interventions.

In summary, this study demonstrates that infertile women undergoing ART exhibit substantial and multidimensional nursing needs, which are significantly influenced by sleep quality, educational level, and treatment stage. Clinical nursing strategies should be individualized and dynamic, integrating psychosocial assessments and stage-specific interventions to optimize patient-centered care (Figure 5). Future studies should consider longitudinal, multi-center designs to further validate these findings and support the development of scalable nursing models tailored to the needs of infertile populations. In summary, this study demonstrates that infertile women undergoing ART exhibit substantial and multidimensional nursing needs, which are significantly influenced by sleep quality, educational level, and treatment stage. Clinical nursing strategies should be individualized and dynamic, integrating psychosocial assessments and stage-specific interventions to optimize patient-centered care. Future studies should consider longitudinal, multi-center designs to further validate these findings and support the development of scalable nursing models tailored to the needs of infertile populations.

## Data Availability

The original contributions presented in the study are included in the article/supplementary material, further inquiries can be directed to the corresponding authors.
